# Synthesis and Application of a Thermoplastic Plate of Poly(lactide-ε-caprolactone) for Radiation Therapy

**DOI:** 10.3390/biom10010027

**Published:** 2019-12-24

**Authors:** Hongli Li, Wenzhi Li, Hongtao Wu, Dengbang Jiang, Mingwei Yuan, Minglong Yuan

**Affiliations:** Green Preparation Technology of Biobased Materials National &Local Joint Engineering Research Center, Yunnan Minzu University, Kunming 650500, Yunnan, China; honglili@vip.163.com (H.L.); liwenzhi0331@126.com (W.L.); wht1755796535@163.com (H.W.); ymujiang@163.com (D.J.)

**Keywords:** poly(lactide-ε-caprolactone) copolymer, triallyl isocyanurate, shape memory, thermoplastic plate, mechanical property

## Abstract

In this study, the poly(lactide-ε-caprolactone) (P(LA-CL)) copolymer is synthesized by ring-opening polymerization with glycol used as a molecular weight regulator to adjust the molecular weight of the polymer. The proton nuclear magnetic resonance spectroscopy and gel permeation chromatography (GPC) results demonstrate that the P(LA-CL) copolymer is successfully synthesized, and that the molecular weight can be controlled by the glycol content. The thermoplastic plate is processed with triallyl isocyanurate as a cross-linking agent by a single-screw extruder followed by γ-ray irradiation. Shape memory test results show that the material had the desired shape memory effect, with deformation recovery rates reaching 100%. After secondary stretching of samples, deformation recovery rates are unchanged. The results of mechanical property measurements indicate that with added lactide, the tensile strength is improved and shore hardness is increased by 20%–30%. Data from clinical trials also reveal that the material has good clinical effects in thermoplastic membrane fixation.

## 1. Introduction

Poly(ε-caprolactone) (PCL) is a biodegradable polyester obtained by the ring-opening polymerization of ε-caprolactone. This semi-crystalline polymer has a degree of crystallinity around 50%, a low glass transition temperature (approximately −60 °C) and a melting point of approximately 60 °C. The PCL chain is flexible, and exhibits high elongation at break, a low modulus, and low processing temperatures [1,2]. This material has good biocompatibility, and can be used to modify other biopolymer materials. For instance, polylactic acid has been modified to increase the toughness and copolymerization of PCL [3], and poly(L-lactic acid) (PLLA) has modified PLLA mechanical properties for use in orthopedic bone repairs [4]. The three complexes were prepared by the vine-twining polymerization method using poly(tetrahydrofuran) (PTHF), poly(ε-caprolactone) (PCL) and poly(l-lactide) (PLLA) as guest polymers to make a film. [5] Degradable polymers, especially polylactic acid (PLA), polyglycolic acid (PGA) and their copolymers, are the most frequently applied coating materials for the stent [6].

The important properties of PCL are shape memory and low-temperature thermoplasticity at 60 °C. PCL has potential applications in the biomedical field, such as in radiotherapy positioning plates, belts, bandages and appliances. However, pure polycaprolactone materials have been found to be imperfect in strength and shape memory.

At present, modification of polycaprolactone is typically achieved through copolymerization or blending with other components to prepare composite materials that meet application requirements. Liang et al. showed that with an increase in the nano-CaCO_3_ weight fraction, the flexural moduli and strength of PCL/nano-CaCO_3_ composites increase roughly linearly and reach a maximum at a filler content of 2%, while the flexural strength of the composites decrease. The flexural moduli and strength of the composites decreased roughly linearly with an increasing PLLA/PCL ratio for the PLLA/PCL/nano-CaCO_3_ composites [7]. To improve the shape memory effect, the PCL were modified by block copolymers containing ethylenic bonds. The network structures of copolymers were then formed by irradiation or reacting with nanogold [8,9,10,11,12]. Organically modified montmorillonite was used to fill PCL to improve crystallinity and thermal stability [13]. Garle et al. reported that functional polycaprolactones synthesized with ε-caprolactone and cinnamate as triblock ABA copolymers with CL as the central block and cinnamate-modified ε-caprolactone as the end blocks showed excellent shape memory effect with a high shape fixation [14].

Previously, our research group developed a polymerization technique with ε-caprolactone and lactide [15,16]. A low thermoplastic plate of pure PCL was prepared by the blending process method with three functions of cross-linking agent. The low thermoplastic plate of pure PCL was industrially produced by the Guangzhou Si’an Company. After more than five years of clinical tests, a better process has been obtained. However, some deficiencies still exist, mainly the low supporting strength and the ineffectiveness of the material with secondary stretching.

As a molecular weight regulator, ethylene glycol is widely used in the synthesis and processing of macromolecules, and there are some reports on the synthesis of polylactic acid, and the previous synthesis experience of our research group can provide guidance. However, there are not many reports on the synthesis of polycaprolactone [17,18].

To rectify the two deficiencies of low thermoplastic plates of pure PCL, the poly(lactide-ε-caprolactone) copolymer is synthesized with ε-caprolactone and lactide using stannous octoate (SnOct) as the catalyst in our study. The novel low thermoplastic plate is prepared with copolymer and triallyl isocyanurate as the cross-linking agent under γ-ray irradiation. In some reports, triallyl isocyanurate was used to cross-link polylactide (PLA) and prepare a composite material [19,20,21,22,23,24,25,26]. However, there are few reports in which PCL composite was prepared using triallyl isocyanurate as the cross-linking agent. In this work, we measure the copolymer structure by use of proton nuclear magnetic resonance spectroscopy (^1^H-NMR) and gel permeation chromatography (GPC). The thermoplastic plate morphology and properties are determined with scanning electron microscopy (SEM), a universal testing machine and differential scanning calorimetry (DSC). Clinical trials of low-temperature thermoplastic plates are carried out in Sichuan Cancer Hospital.

## 2. Materials and Methods 

### 2.1. Materials

L-lactide was purchased from Purac Company, Netherlands. The ε-caprolactone was provided by China Asset Management of Petrochemical Group Co. Ltd. The SnOct and glycol were purchased from Aladdin Reagent Ltd, Shanghai, China. Triallyl isocyanurate (TAIC) was purchased from Si’an Company, Guangzhou, China. All chemicals were used as received.

Collector magnetic agitator(DF-101S) and Circulating water vacuum pump(SHZ-D) were purchased from Gong yi Yuhua instrument co. Ltd. Precision booster electric mixer(JJ-1) was provided by Aohua instrument co. Ltd, Changzhou, China.

### 2.2. Methods

#### 2.2.1. Synthesis of Poly(lactide-ε-caprolactone) Copolymer 

The opening ring polymerization of different weight ratios of ε-caprolactone (70%, 80%, and 90%) and lactide (with a total monomer weight of 400 g) was performed in a 1000-mL three-necked flask equipped with a magnetic stirrer, using 0.15% (*g*/*g*) SnOct as a catalyst. The reactants were filled with high purity argon, degassed in vacuum three times, and then sealed. Glycol as a molecular-weight regulator was added into the reaction system at dosages of 0, 0.0625, and 0.125% (*g*/*g*).

**Figure 1:**
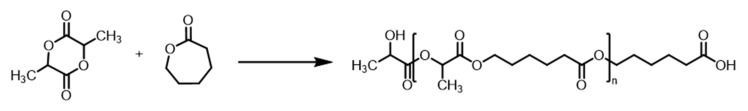
The synthesis of copolymers of lactide and caprolactone.

**Figure 2:**
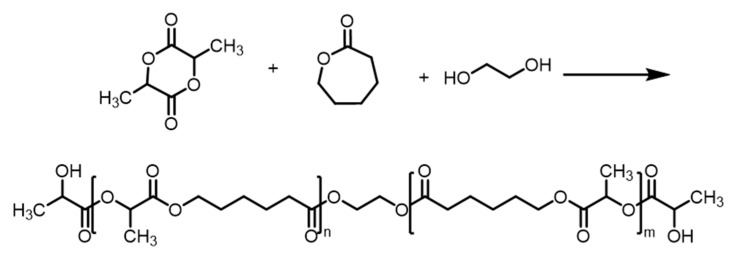
The synthesis of copolymers of lactide and caprolactone with ethylene glycol.

Under argon gas protection and a polymerization temperature of up to 180 °C, the reaction time was controlled at 4 h. Following the reaction, the system was cooled to 100 °C. The polymer was poured into a water tank to cool and then pulled into thin strips. The strips were cut into small copolymer pellets by hand. The synthesis mechanisms of copolymers are shown in Figure 1 and Figure 2. To examine the effects of the monomer ratio, we prepared a number of synthetic copolymers from A to I according to the ratio of ε-caprolactone and lactide. The pure poly(ε-caprolactone) (PCL) is identified as Sample J. Specific synthesis conditions are shown in Table 1.

#### 2.2.2. Preparation of Thermoplastic Plates

Thermoplastic plates were prepared by a single-screw extruder (LSJ-20, Shanghai, China). The poly(lactide-ε-caprolactone) (P(LA-CL)) copolymer and triallyl isocyanurate at a ratio of 0.2% copolymer were added to the extruder. A smooth, thermoplastic plate was prepared when the processing temperatures of each section were 70, 70, 80, and 70 °C at a 40 r/min screw speed. At these settings, the melt of the extruded material added by a single screw is pressed out and cooled by a three-roll machine to obtain a plate with a thickness of 2 mm. Finally, we cut, punch and pack according to design requirements.

The packaged sheet is entrusted to a radiation plant to undergo r-ray irradiation, and the irradiation measurement is 5–10 KGY, and the size of the irradiation measurement is determined according to the use requirements of the low temperature thermoplastic board.

#### 2.2.3. Measurements

##### ^1^H-NMR Measurements

The ^1^H spectra of copolymers were recorded with a nuclear magnetic resonator (NMR) (Bruker Avance III 400 MHz, Switzerland). Deuterated chloroform (CDCl_3_) was used as the solvent at 25 °C and the chemical shifts were given with respect to tetramethylsilane. ^1^H spectra were obtained from 16 scans.

##### GPC Measurements 

The molecular weights and molecular distributions of PCL and P(LA-CL) with different ratios were determined by gel permeation chromatography (GPC) with a Waters Associates model ALC/GPC 244 apparatus at 40 °C with a differential refractometer as the detector, tetrahydrofuran (THF) as the solvent, and calibration with polystyrene standards. Three specimens were tested under each condition.

##### SEM Measurements

The cross-sectional morphology of thermoplastic plate was observed directly by a scanning electron microscope (SEM) (Quanta200, FEI, Hillsboro, USA) without sputter coating, but with a conducting matter. The sample was first frozen in liquid nitrogen and then lyophilized at −47 °C.

##### Shape Memory Process Measurements

The thermoplastic plate was heated in 60 °C water bath for 10 minutes, and then it was stretched by a certain length. The original and stretched lengths were recorded. The stretched sample was heated again with the same time and temperature and the recovering length was measured. After this, the recovered sample was re- placed heated in 60 °C water and repeat the above shape memory experiment Mechanical Property Measurements.

Tensile tests were conducted using a universal testing machine (GMT-400; Shanghai, China) using the national standard GB/T 1040. All samples were cut to 100 mm × 20 mm. The tensile speed was 10 mm/min. All reported results are the averages of at least three test specimens. The elongation at break of samples can be calculated according to equation (1), where Eb(%) is the elongation at break, L (mm) is the distance between the lines when the sample is broken and L_0_ (mm) is the original line distance of the sample.

##### Shore Hardness Measurements

The shore hardness was measured with a digital shore durometer (SAUTER HDD100-1; Germany). Each set of tests was performed in triplicate.

##### DSC Measurements

Differential scanning calorimetry (DSC) was performed using a TA Instruments DSC-200PC (Netzsch, Germany). An empty pan was used as a reference. After being dried in a vacuum oven at 40 °C for 48 h, circular samples were cut from the plates and accurately weighed (5 mg) into aluminum pans. DSC measurements were carried out under nitrogen flow from 30 °C to 200 °C at a heating rate of 5 °C/min.

##### Clinical Trials of P(LA-CL) Low-Temperature Thermoplastic Plates

**Figure 3:**
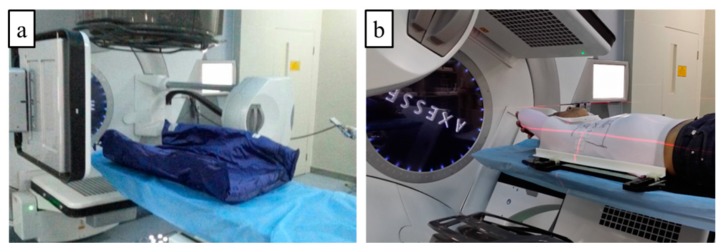
Fixation of chest tumor radiotherapy (**a**) Vacuum pad fixation, (**b**) Thermoplastic membrane fixation).

During the clinical testing phase, 60 patients with breast cancer received radiation therapy in the Department of Radiation Oncology of Sichuan Cancer Hospital and were confirmed by pathology. Inclusion criteria included patients aged older than 30 years and weighing more than 38 kg. Of 60 patients, 43 were male and the patients were randomly enrolled into two groups: 30 patients in the vacuum pad fixation group identified as Group 1 and 30 patients in the thermoplastic membrane fixation group identified as Group 2. Vacuum pad fixation and thermoplastic membrane fixation are shown in Figure 3. The vacuum pad fixation group included 15 lung cancer and 15 esophageal cancer patients consisting of 19 men and 11 women. The thermoplastic membrane fixation group included 1 thymic carcinoma, 17 lung cancer and 12 esophageal cancer patients consisting of 15 men and 15 women. All of the patients gave their informed consent before treatment, which was in accordance with the Declaration of Helsinki and also approved by the Ethics Committee of Sichuan Cancer Hospital.

**Figure 4:**
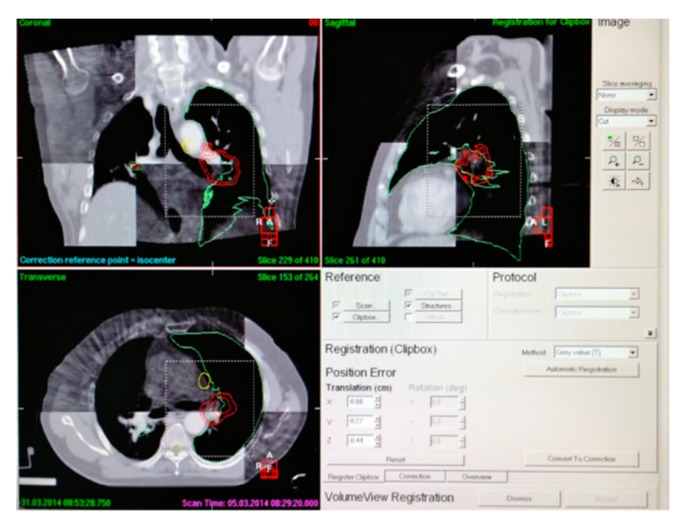
Cone beam computed tomography (CBCT) image calibration and positioning error calculation.

Two groups of patients were treated with intensity-modulated radiation therapy combined with computed tomography (CT) simulation to accurately locate tumors. The image data obtained were used in treatment planning. According to the requirements to determine the positioning center, under the CT simulator will be Move the reference center to the positioning center. Using cone beam CT (CBCT) scans shown in Figure 4, three translational setup errors in the lateral (X), cranial-caudal (Y) and anterior-posterior directions (Z), were obtained. The U, V and W rotation setup errors were obtained by image analysis software to determine the corresponding X, Y and Z values.

##### Statistical Analysis

Data were analyzed according to the mean and standard deviation of three test replicates for each sample. The statistical analysis was performed by analysis of variance (ANOVA) using SPSS version 13.0 at a significance level of *p* < 0.05.

## 3. Results and Discussion

### 3.1. Characterization of Copolymers

**Figure 5:**
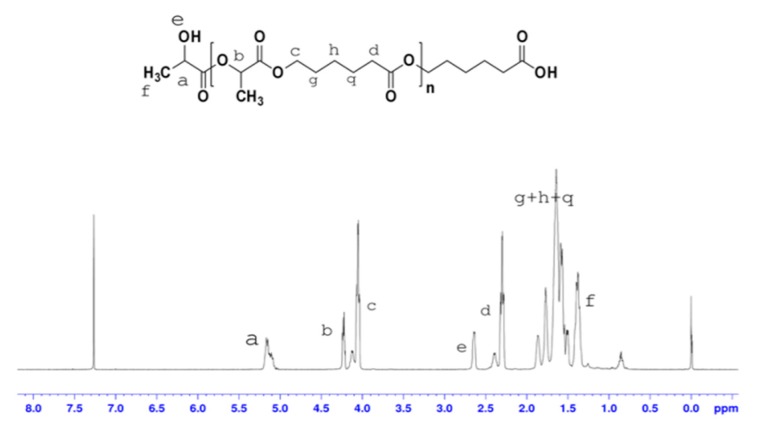
Protonic nuclear magnetic resonance (^1^H-NMR) spectrum of the poly(lactide-ε-caprolactone) (P(LA-CL)) copolymer without glycol.

**Figure 6:**
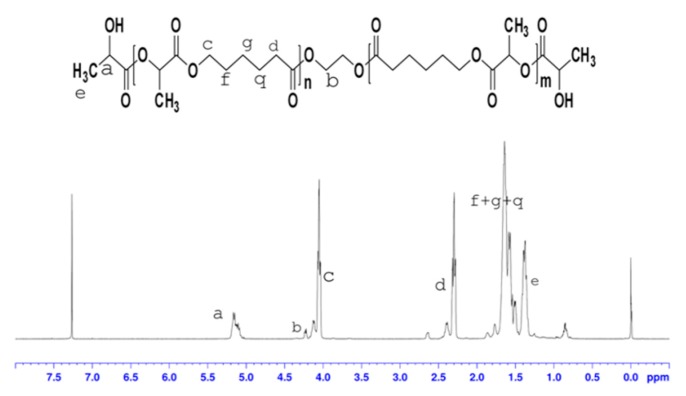
^1^H-NMR spectrum of the poly(LA-CL) copolymer with glycol.

The conversion and yield of a polymer is an important indicator of whether the polymer can be mass produced. As shown in Table 2, the conversion and yield of nine copolymers were more than 90%. Comparison with similar literature indicates that yields and conversion rates have been greatly improved [14], and that the yields obtained in our study are able to meet production requirements. To confirm their structures, copolymers were characterized with ^1^H-NMR. Figure 5 and Figure 6 show ^1^H-NMR spectra of the poly(LA-CL) copolymer without glycol and poly(LA-CL) copolymer with glycol, respectively. In Figure 5 and Figure 6, the ^1^H-NMR spectrum of lactide shows peaks at 1.44 and 5.17 ppm, which are assigned to the methyl and methine protons of the PLA chains, respectively. The ^1^H-NMR spectrum of caprolactone shows peaks at 1.52, 2.35, and 4.12 ppm, which are assigned to methylene protons from (CH_2_)_3_, CO–CH_2_, and CH_2_–O groups of the PCL chains, respectively. These results are consistent with those previously reported [15,16].

**Figure 7:**
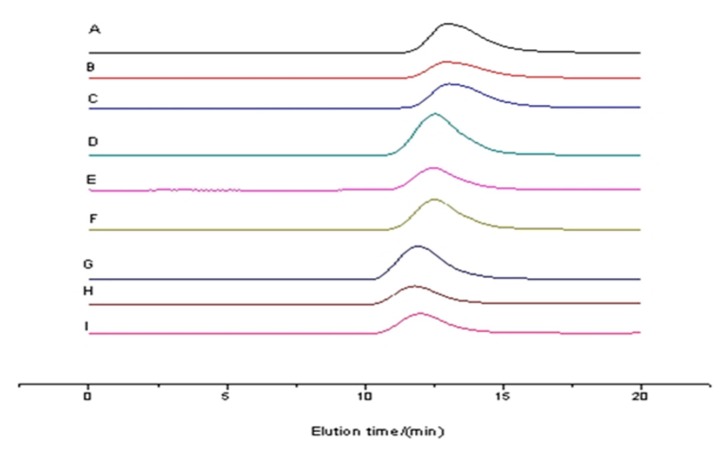
Gel permeation chromatography (GPC) curves of copolymers.

Figure 7 shows that the gel permeation chromatography (GPC) curves of the copolymer samples each consist of only a single molecular weight distribution peak. These results indicate that the target product is a copolymer rather than a homopolymer blend. It can be seen in Table 3 that molecular weights increased with a decline in glycol content. The effect of glycol on molecular weight is significant; the molecular weight reached approximately 20 kDa in the absence of glycol and was significantly reduced with its addition. Because the molecular weight can affect the properties and use of polymers, selecting a polymer with an appropriate molecular weight is also a purpose of our study.

### 3.2. Characterization of Thermoplastic Plates 

**Figure 8:**
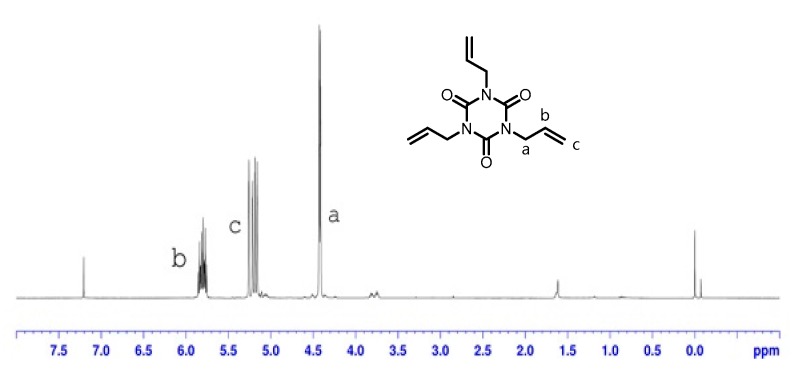
^1^H-NMR spectrum of triallyl isocyanurate (TAIC).

**Figure 9:**
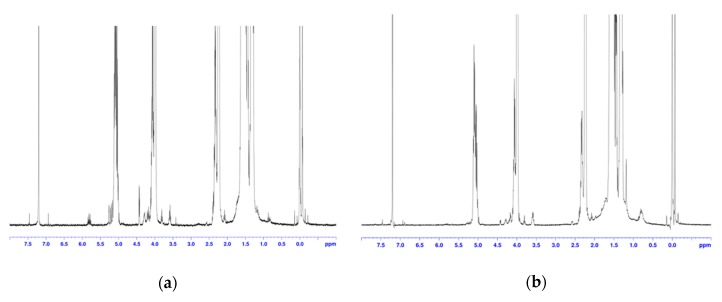
^1^H-NMR spectrum of a thermoplastic plate without irradiation (**a**) ^1^H-NMR spectrum of a thermoplastic plate with irradiation (**b**).

In polymer material processing and modification, triallyl isocyanurate (TAIC) is one of the most commonly used cross-linking agents in industrial processes. TAIC has been reported in literature as an agent for cross-linking PLA in the preparation of cross-linked composites. However, the preparation of PCL composite materials using TAIC as a cross-linking agent is typically not reported. We have thus used TAIC to cross-link P(LA-CL) copolymers and prepare composite materials in our study. Figure 8 is the ^1^H-NMR spectrum of TAIC and Figure 9a,b are the ^1^H-NMR spectra of thermoplastic plates without irradiation and with irradiation, respectively. Comparison of Figure 9a,b indicate an absence of TAIC characteristic chemical shifts after irradiation. This suggests that the TAIC has reacted with P(LA-CL). To confirm that TAIC acted as a cross-linking agent, the properties of composite materials are examined in the following subsection.

### 3.3. Mechanical Property Analysis of Thermoplastic Plates

**Figure 10:**
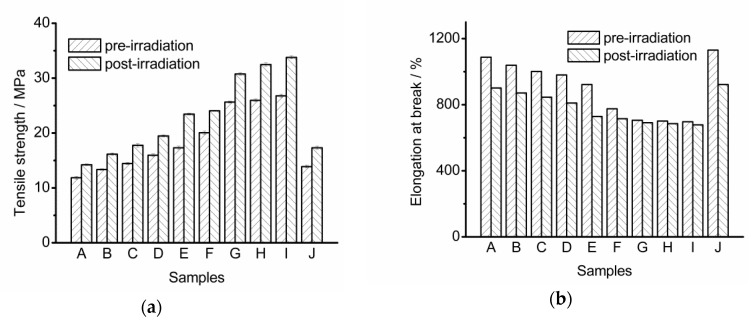
Tensile strength of materials pre- and post-irradiation (**a**) Elongation at break of materials pre- and post-irradiation (**b**).

The tensile strength and elongation at break describe how the mechanical properties of materials are related to their chemical structure [11]. The tensile strength and elongation at break of low thermoplastic plates pre- and post-irradiation are presented in Figure 10a,b. All reported results are the averages of at least three test specimens. Lactide is a rigid segment. As the content of lactide increases, the stiffness of the copolymer is increased, so the tensile strength is increased. When the lactide content was more than 20%, the tensile strength of the composite samples was higher than that observed for pure PCL. Comparison of pre- and post-irradiation samples also indicated that tensile strength was enhanced by irradiation.

This is consistent with the change in morphological structure upon irradiation as proven by scanning electron microscopy (SEM). In contrast, the elongation at break decreased with an increase in lactide content.

**Figure 11:**
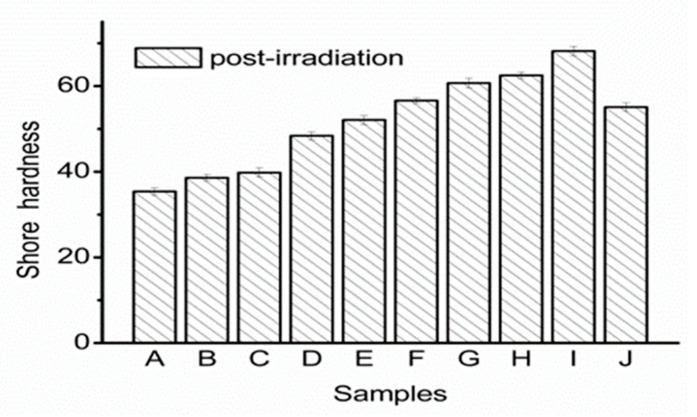
Shore hardness of composite materials.

For the shape memory of a low-temperature thermoplastic plate prepared with PCL composite material, the shore hardness is a more intuitive index for practical applications. According to clinical expert feedback, the shore hardness of low-temperature thermoplastic plates in the current market can reach up to 40 D. With the clinical requirements, normally, it needs to be stretched 1–3 times in local or overall during the use. After stretching, the material becomes longer and thinner, so the model after molding becomes very soft, and the supporting force is very weak, which seriously affects the use effect. The hardness of low thermoplastic plates is hoped to improve to about 50 D [27,28], and Figure 11 shows the shore hardness of P(LA-CL) thermoplastic plates after irradiation. The shore hardness improved with an increase in lactide content and molecular weight; at an Mw of 10.5 kDa, the shore hardness reached 48.4 D. The data shows that the hardness of the three groups A, B and C, is less than 40 D, while the hardness of the three groups G, H and I is too large, and the deformation temperature is too high. It is not conducive to clinical application when the shore hardness is too high. Therefore, Samples D, E and F are considered ideal thermoplastic plate materials.

### 3.4. DSC Analysis of Thermoplastic Plates

**Figure 12:**
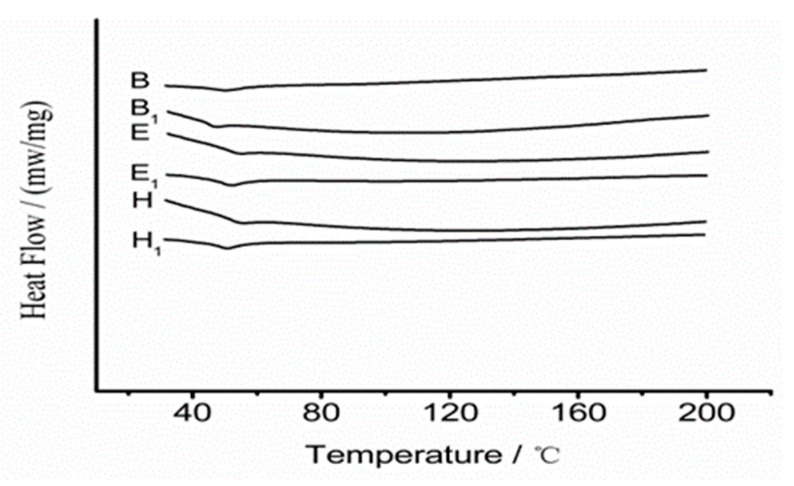
Differential scanning calorimetry (DSC) curves of P(LA-CL) composite materials (B, E, and H without irradiation and B1, E1, and H1 with irradiation).

Based on the tensile test, shore hardness test, morphology test and molecular weight study, samples synthesized with a CL:LA ratio of 8:2 were chosen for studies with DSC. Figure 12 shows the DSC curves of P(LA-CL) composite materials with and without irradiation. As shown in Figure 12 and Table 4, the melting temperature was improved by irradiation. The thermal stability also increased as the molecular weight increased. At molecular weights above 11.1 kDa, the Tm showed little increase with further increases in molecular weight. The following clinical experiments were thus conducted using the composite material from Sample E. Since the radiation dose we use is very low, it does not alter crystallization behavior [29,30].

### 3.5. SEM Analysis of Thermoplastic Plates

**Figure 13:**
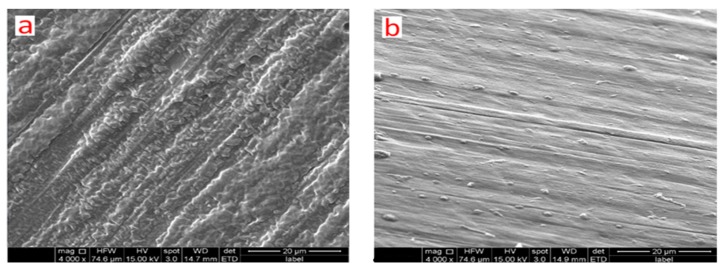
The cross-sectional morphologies of thermoplastic plates (**a**) pre-irradiation sample, (**b**) post-irradiation sample.

Based on the results of the tensile test, shore hardness test, morphology test and molecular weight test, Figure 13 shows the cross-sectional morphologies of pre- and post-irradiation samples. The pre-irradiation sample consisted of a small block structure that exhibited structural relaxation. However, the flexible structure of the thermoplastic plate disappeared upon exposure to radiation, and its structure became dense and rigid. This change in structure may be explained by the effect of radiation in inducing reactions with the cross-linking agent that could affect the material properties.

### 3.6. Shape Memory Process Analysis of Thermoplastic Plates

**Figure 14:**
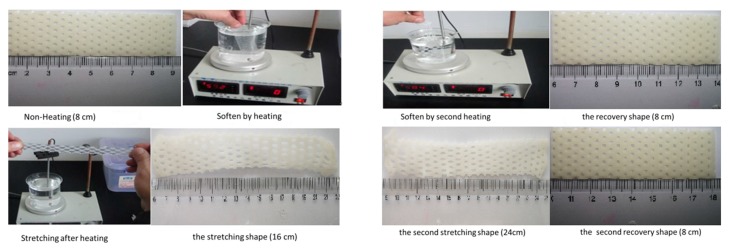
Shape memory process analyses of thermoplastic plates.

Based on the results of the tensile test, shore hardness test, morphology test and molecular weight test, we choose group E for the shape memory test. The material shown in Figure 14 exhibited the desired shape memory effect where the deformation recovery rate had substantially reached 100%. The deformation recovery rate was essentially unaffected by secondary stretching. Despite a secondary stretching length of 1.5 times the first stretch, the thermoplastic plate returned to its initial length upon heating. Thus, a composite material with shape memory has been achieved in this study.

### 3.7. Clinic Data Analysis of Thermoplastic Plates 

Clinical tests were processed in the Sichuan Cancer Hospital in Sichuan Province. Groups 1 and 2 are trialed vacuum pad fixation and thermoplastic membrane fixation, respectively. The setup errors are shown in Table 5. The results revealed that setup errors with thermoplastic membrane fixation were less than errors for vacuum pad fixation in all directions with P values lower than 0.05.

## 4. Conclusions

In our study, PCL was modified with biocompatible and biodegradable PLA, and glycol was used as a molecular weight regulator to adjust the molecular weight of the polymer. The results demonstrated that the P(LA-CL) copolymer was successfully synthesized with the conversion and yield of nine copolymers at more than 90% using our synthesis method. To get a better shape memory function, we used TAIC with three terminal alkenyl functional groups as a cross-linking agent to prepare the composite material. The thermoplastic plate was processed by a single-screw extruder, and then placed under γ-ray irradiation. Shape memory and performance test results showed that the material has the desired shape memory effect with a deformation recovery rate reaching 100%. After the secondary stretching of samples, the deformation recovery rate remained unchanged. With the added lactide, the tensile strength was improved and shore hardness increased by 20–30%. Data from clinical trials reveal that the material has good clinical effects in thermoplastic membrane fixation. In summary, the low thermoplastic plate of the P(LA-CL) copolymer has very good application prospects in radiotherapy positioning.

## Figures and Tables

**Table 1:** Monomer ratios of copolymer samples.

Sample	m_ε-caprolactone_:m_lactide_	Total Monomer Weight (g)	Glycol (g)	SnOct (g)
A	9:1	400	0.5	0.6
B	8:2	400	0.5	0.6
C	7:3	400	0.5	0.6
D	9:1	400	0.25	0.6
E	8:2	400	0.25	0.6
F	7:3	400	0.25	0.6
G	9:1	400	0	0.6
H	8:2	400	0	0.6
I	7:3	400	0	0.6
J	10:0	400	0	0.6

**Table 2:** Conversion and yield of copolymers.

Sample	m_ε-caprolactone_:m_lactide_	Total Monomer Weight (g)	Conversion (%)	Yield (%)
A	9:1	400	97.1	90.7
B	8:2	400	96.8	94.2
C	7:3	400	96.4	96.3
D	9:1	400	96.7	94.9
E	8:2	400	97.7	93.7
F	7:3	400	97.4	92.4
G	9:1	400	95.8	91.9
H	8:2	400	96.2	94.6
I	7:3	400	95.6	95.8

**Table 3:** Molecular weights of copolymers.

Sample	*M_n_* (kDa)	*M_w_* (kDa)	PDI
A	4.2	6.3	1.498
B	4.4	6.5	1.475
C	4.0	6.0	1.490
D	6.5	10.5	1.611
E	7.49	11.1	1.475
F	7.09	11.0	1.505
G	11.05	19.0	1.721
H	12.44	21.0	1.687
I	10.94	17.3	1.581

**Table 4:** Melting temperatures of P(LA-CL) composite materials.

Sample	Tm (°C)	Remark
B	47.4	pre-irradiation
B1	50.1	post-irradiation
E	52.2	pre-irradiation
E1	55.2	post-irradiation
H	50.9	pre-irradiation
H1	56.3	post-irradiation

**Table 5:** Comparison of setup error results from vacuum pad fixation (Group 1) and thermoplastic membrane fixation (Group 2).

Project	Group	Setup Error $\left(\bar{x}\pm s\right)$	Median	Maximum	Minimum	*p* Value
X (mm)	1	0.81 ± 3.78	1.33	8.70	−8.70	0.02
	2	−1.35 ± 0.45	−0.30	3.20	−1.90	
Y (mm)	1	1.75 ± 4.55	2.15	10.00	−8.75	0.01
	2	1.15 ± 2.55	1.60	7.55	−5.00	
Z (mm)	1	2.45 ± 2.20	2.15	7.35	−3.30	0.005
	2	2.35 ± 0.35	1.25	5.00	−2.75	
U (mm)	1	0.60 ± 0.65	0.65	2.10	−1.80	0.01
	2	0.08 ± 0.32	0.00	0.65	0.70	
V (mm)	1	0.35 ± 1.40	0.45	2.55	−3.85	0.03
	2	−0.10 ± 0.50	0.00	1.30	−2.00	
W (mm)	1	0.45 ± 0.90	0.40	3.45	−1.35	0.01
	2	0.15 ± 0.32	0.08	1.25	−1.25	
